# Caloric and Lipid Profiles during Pregnancy in a Socio-Culturally Diverse Society

**DOI:** 10.3390/foods12051111

**Published:** 2023-03-06

**Authors:** Elisabet Fernández-Gómez, Miriam Mohatar-Barba, María López-Olivares, Trinidad Luque-Vara, María Angustias Sánchez-Ojeda, Adelina Martín-Salvador, Carmen Enrique-Mirón

**Affiliations:** 1 Department of Nursing, Faculty of Health Sciences, Melilla Campus, University of Granada, C/Santander s/n, 52001 Melilla, Spain; 2 Melilla Campus, University of Granada, C/Santander s/n, 52001 Melilla, Spain; 3 HUM-613 Research Group, Department of Inorganic Chemistry, Faculty of Health Sciences, Melilla Campus, University of Granada, C/Santander s/n, 52001 Melilla, Spain

**Keywords:** caloric profile, lipid profile, diet quality, culture, pregnancy, nutrition

## Abstract

This research analyzes the determining factors in diet quality among the Spanish pregnant population with the aim of promoting healthier eating habits and preventing the development of non-communicable diseases. It is a diagnostic, non-experimental, cross-sectional, and observational study, with correlational descriptive methodology, and 306 participants. The information was collected using the 24 h dietary recall. Various sociodemographic factors that influence diet quality were analyzed. It was found that pregnant women consume too much protein and fat, score high in SFA consumption, and do not achieve the CH recommendations, consuming twice as much sugar. Carbohydrate intake is inversely related to income (β = −0.144, *p* < 0.005). Likewise, protein intake is linked to marital status (β = −0.114, *p* < 0.005) and religion (β = 0.110, *p* < 0.005). Finally, lipid intake appears conditional upon age (β = 0.109, *p* < 0.005). As regards the lipid profile, a positive association is only observed with age and MFA consumption (β = 0.161, *p* < 0.01). On the other hand, simple sugars are positively related to education (β = 0.106, *p* < 0.005). The results of this research show that the diet quality of pregnant women does not meet the nutritional recommendations established for the Spanish population.

## 1. Introduction

At present, diet is considered one of the factors of greatest influence on well-being, health, and life quality, thus showing a direct action on the morbidity and mortality of a determined population. Hence, a healthy diet is one of the most important aspects for improving health [1].

Noncommunicable diseases (NCD) are the main cause of death and disability in women worldwide, including women at a reproductive age [2]. The Sustainable Development Agency (SDA) includes specific targets on maternal health and NCD, such as a reduction in the global maternal mortality rate to 70 deaths for every 100,000 live births, and a reduction by one-third in premature mortality due to NCD [3].

NCD, including cardiovascular diseases (CVD), cancer, chronic respiratory diseases, and neurodegenerative diseases, are the main cause of morbidity and mortality in the world. Among the main risk factors, we can highlight those aspects relating to lifestyle, such as an unbalanced diet, obesity, physical inactivity, emotional state, and quality of life as well as smoking and alcohol consumption [4,5,6]. Nevertheless, excess weight and obesity continue to rise each year due to multiple factors, including poor eating habits [7,8,9], characterized by high levels of processed meats and fats, saturated fats, refined grains, salt and sugars, and a lack of fresh foods, fruit, and vegetables [10].

At the end of the 19th century, the Spanish diet gradually covered nutrient and energy requirements, being more costly in minors, adult women, and pregnant women. At the end of the 20th century and the beginning of the 21st, as has occurred in other countries, energy intake has increased in an excessive and unbalanced manner causing deficiencies in the main micronutrients [11].

The gestation period is a critical time for establishing the risks of chronic diseases in offspring [12]. Nutrition plays a key role during this period of development and, since it is a determining factor of risk throughout life, it is a modifiable risk factor. Although the World Health Organization (OMS) provides guidelines for prenatal care [13], there is a lack of comprehensive guidelines detailing the nutritional requirements of women during reproduction, from preconception through pregnancy and breastfeeding [14].

Pregnancy is a vulnerable stage where the evidence on maternal nutrition shows the importance it exercises on the mother’s health and on healthy fetal growth and development [15]. An inadequate nutritional intake during this stage of life may have negative consequences for health in the short and long term, both for the mother [16] and for the child [17] such as premature births or miscarriages [18], hypertensive disorders [19], obesity or diabetes in childhood [20,21,22], alterations in fetal growth [23], and susceptibility to allergies and bacterial infections [24] among others. Nevertheless, a healthy diet before and during pregnancy is associated with a lower risk of all these diseases [25,26,27,28]. 

Some studies indicate that Spanish women do not meet the dietary recommendations of scientific societies [29,30]. This failure is related to pregnant women’s socioeconomic level, culture, age, and tobacco and alcohol consumption [31,32,33], among different factors. Therefore, culture is a factor that influences dietary habits. The fact is that the frequency of daily food intake or the quantity or the type of food consumed will depend more on culture than the availability of the food in itself [34,35].

The city of Melilla, where this study is performed, is a border city with Morocco, which contributes to the city’s multiculturality. This proximity means that many Moroccans cross the border to seek healthcare [36]. Melilla is, therefore, an optimum scenario for studying the social and cultural differences in relation to eating habits during pregnancy.

Hence, a general objective of this study is to analyze dietary quality in pregnant women in the multicultural city of Melilla, as well as the factors that may influence it with the aim of promoting healthy eating habits at this stage.

## 2. Materials and Methods

### 2.1. Study Design and Participants

It is a diagnostic, non-experimental, cross-sectional, and observational study, with correlational descriptive research methodology.

The sample was selected by convenience probability sampling from the populational data on pregnant women collected in the public health system of the city of Melilla in the last 18 years. The sample is formed of 306 pregnant women, with an average age of 29.92 (5.51) years old, with the minimum age being 18 and a maximum age of 43; specifically, 196 (64.1) were born in the city of Melilla. The characteristics of the sample such as residence, place of birth, number of children, marital status, education, and income are shown in Table 1.

### 2.2. Instruments and Procedure

The 24 h dietary recall by Rodríguez et al. [37] was used to determine diet quality. It is a questionnaire completed by the participants recording the number of grams of food ingested during the previous day (breakfast, lunch, afternoon snack, and food intake between meals) after providing them with the appropriate explanation to estimate said amounts in order to increase result reliability [38]. Tables were used with images of representations of food and drinks with various sizes and grams to facilitate data collection.

This questionnaire not only obtains in detail the quality and quantity of the food and drink (grams), but also details the culinary process and places emphasis on the quantity and quality of bread, oil, and sugar.

The participants completed the questionnaires in-person after written informed consent was provided. These data were gathered between March and December 2021.

### 2.3. Statistical Analysis

The data obtained were analyzed with the statistical program SPSS in its version 26.0 (International Business Machines Corporation (IBM), Armonk, NY, USA).

The basic statistics were used, according to the nature of the variables, for the descriptive analysis. Thus, for the quantitative variables the measures of central tendency (mean, median, mode), dispersion (typical deviation), and position (distribution limits) measurements were used whilst absolute and relative frequencies (percentages) were used for the qualitative variables.

Non-parametric tests were used for the interferential analysis according to the values presented by the Kolmogorov–Smirnov test. The chi-squared test was used for the comparison of proportions and *p* < 0.05 was considered a value of statistical significance. Likewise, the Mann–Whitney U test and the Kruskal–Wallis test were used to relate diet quality with sociocultural factors depending on the number of categories of independent variables.

Three multiple regression models were performed with the independent variables dichotomized with the aim of verifying the degree of determination that the sociodemographic variables may have on the caloric, lipid, and simple sugar consumption profiles. An Ordinary Least Squares (OLC) analysis was performed to compare the dependent variables (caloric profile, lipid profile, and simple sugar consumption) with the rest of the study variables; standardized and non-standardized regression coefficients were also obtained (β). 

The data provided by the 24 h dietary recall in the form of food were transformed into energy intake, consumption of macronutrients (carbohydrates, lipids, and proteins), micronutrients (vitamins and minerals), and plant fiber using the IENVA dietary calculator (https://calcdieta.ienva.org/tu_menu.php, accessed on 3 January 2023) with the advice of a nutritionist. The grams of daily consumption of each immediate principle were compiled, as well as the sugars and types of fatty acids, it was later multiplied by the kcal that each one of them provides, and to calculate the percentage of the total caloric value (TCV), it was multiplied by 100 and divided by the total kcal ingested.

### 2.4. Ethical Considerations

This research was governed by the ethical principles of the Declaration of Helsinki.

The participants were informed of the study characteristics, as well as its objectives, and agreed to take part voluntarily. Formal consent was requested by signing the informed consent.

## 3. Results

With respect to the intake of macro and micronutrients, the nutritional requirements varied for pregnant women depending on whether they were in the first or second half of pregnancy. For this reason, the data referring to nutrient intake are shown distributed in line with this nutritional parameter. Table 2 shows the median and interquartile range values related to the intake of energy, macronutrients, fiber, and cholesterol. 

The caloric and lipid profiles are found in Table 3. With respect to the caloric profile for the total sample, proteins provide 16.29% of the energy consumed, 45.46% corresponds to carbohydrates (18.6% in the form of simple sugar), and the remaining 38.36% is provided by lipids. In terms of the lipid profile, the energy intake of saturated fats (SFA) for the total sample is 11.53%, monounsaturated fats (MFA) provide 18.26%, and polyunsaturated fats (PFA) 4.93%. There are significant differences between the two gestation periods for lipids (*p* = 0.006) in the case of the caloric profile and for MFA (*p* = 0.010) and PFA (*p* = 0.018).

Table 4 shows how the caloric profile is distributed within the limits established according to the nutritional targets established for the Spanish population [39]. In this regard, it is observed how more than half of the participants in both periods have a higher protein consumption (for 54.5% and 57.8%, in the first half of pregnancy and in the second, respectively, the proteins make up a TCV contribution greater than 15%). Likewise, for 71% and 65.8%, the fats were greater than 35% of the VCT. With respect to carbohydrates, only 16.6% and 21.1% of the pregnant women corresponding to the first and second gestation period, respectively, follow the recommendations (between 50 and 55%). In relation to the aggregate sugars, it should be highlighted that 87.6% and 91.9% of pregnant women belonging to the first or second gestation period consume more than 10% of the recommended sugars.

As regards the lipid profile, over half of the participants, belonging to both gestation groups, tend to consume more than 10% of SFA (Table 5).

Table 6 sets down the consumption of micronutrients (minerals and vitamins) in both gestation periods. It should be stressed that the mineral intake does not meet the recommendations with respect to calcium, iron, zinc, magnesium, and potassium. On the other hand, as regards the vitamins, the intake is not met for FA and vitamins A, D, and E. 

The results of the regression models performed considering the caloric profile as dependent variables and the sociodemographic variables as independent variables are shown in Table 7. Carbohydrate intake is inversely related to income (β = −0.144, *p* < 0.005), so with lower incomes there would be greater carbohydrate consumption. Likewise, protein intake is linked to marital status (β = −0.114, *p* < 0.005) and religion (β = 0.110, *p* < 0.005) since pregnant women with a partner and those who are Muslim consume the least amount of protein. Finally, lipid intake appears conditional upon age (β = 0.109, *p* < 0.005) with the oldest women consuming the largest amount of this macronutrient.

Table 8 shows the results of the regression performed on the lipid profile as dependent variables, and the sociodemographic variables as independent variables. As regards the lipid profile, a positive association is only observed with age and MFA consumption (β = 0.161, *p* < 0.01); i.e., the youngest pregnant women consumed the least MFA. On the other hand, simple sugars are inversely related to education (β = 0.106, *p* < 0.005). The women with the lowest education are those who consume the highest amount of simple sugars.

## 4. Discussion

An imbalanced caloric and lipid profile is observed in this research. Over half of the participants show an intake exceeding the protein recommendations, which also occurs in other studies [41,42]. Carbohydrate intake was below the recommendations and the total fat intake was exceeded according to the dietary references, coinciding with other studies [29,43,44,45]. Nevertheless, although these studies did not differentiate between the types of fats consumed in the diet, it is known that ϖ-3 fatty acids during pregnancy improve infant cognitive development [46] and prevent allergic diseases [47]. However, the fact that the total fat intake exceeds the recommendations may contribute to an unhealthy increase in maternal weight. This is associated with a higher risk of preeclampsia, gestational Diabetes Mellitus, macrosomia, congenital anomalies, and newborns with low birth weight and maternal mortality [48].

The group studied ingests an average of 1891.35 ± 529.18 kcal/day, practically coinciding with Izquierdo Guerrero [40], which introduced an average of 1984.75 ± 579.84 kcal/day. The average energy intake in pregnant women during the first gestational period is 1767 Kcal/day and of 1898 Kcal/day in the second. Therefore, it can be observed that pregnant women do not reach the energy recommendations [39].

Around 90% of pregnant women eat more sugar than they should. The high consumption of commercial juices, pastries, sweets, and ice cream is responsible for this; our data are similar to the ANIBES study [49]. A higher consumption of sugars and fatty acids in pregnancy is associated with high adiposity in the offspring [50,51,52].

With respect to sugars, the WHO [53] recommends decreasing consumption below 10% of the total energy intake, since it causes an increase in weight and tooth decay. A consumption below 5% would give rise to health benefits.

Furthermore, the participants’ lipid profile has a high energy intake through SFA, as occurs in other studies with similar groups [41,54,55]. MFA and PFA, however, do not reach the recommended consumption percentage. Likewise, the study by Ortega Anta et al. [56] highlights the low PFA consumption, recommending the increase in consumption of fish and/or food enriched with PFA to achieve health benefits.

According to the FEN (Spanish Nutritional Foundation) [57] women of fertile age should take care of their diet, not excluding any essential nutrients so that when they become pregnant, they do not undergo additional nutritional risks. AGP-3 should be provided daily in the diet through fish or nuts, among other foods containing contain them.

An intake of 22–25 g of fiber per day is recommended in women. Unfortunately, in Europe, these recommendations are not reached and very few countries offer guidance on the sources of food that contain fiber to achieve a suitable intake [58]. There are countries, such as the Scandinavian countries, which recommend a higher intake of wholegrains, approximately 75 g per day [59]. Fiber consumption in this study is considerably less than the recommendations, 12–15 g per day, far from the reference data. Furthermore, Carbajal-Azcona [60] adds the intake of around 35 g/day of fiber and moderating sugar intake in his general nutritional advice for pregnant women.

A meta-analysis of the dietary data obtained in several developed countries informed that pregnant women have difficulties in following the national dietary guidelines for macro and micronutrients [61]. The same occurs in the research presented here: they do not meet the intake recommendations of the minerals, calcium, iron, magnesium, zinc, and K and vitamins A, D, and E, and FA, coinciding with Kocyłowski et al. [62], and with Rodríguez-Bernal et al. [29] where an insufficient intake of FA, iron, and vitamin E was shown. Likewise, the results shown coincide with other studies where a lack of folates, vitamin D, calcium, iron, iodine, zinc, and vitamins of the group [63,64] is verified.

Adequate iron intake during pregnancy may reduce the risk of anemia, newborns with low weight, and premature births [65,66]. It is also important to maintain an adequate calcium intake since it helps reduce the risk of preeclampsia [67].

The Expect I study, based on calcium intake in the diet and the use of supplements in pregnant women in the Netherlands, concluded that 42% of pregnant women had an inadequate calcium intake and that supplements are frequently used, but the majority do not contain sufficient quantities to remedy this inadequate intake [68]. Likewise, calcium consumption in this study is fairly insufficient, lacking around 400–500 mg daily.

FIGO highlights the importance of a healthy and varied diet, with supplements or fortified foods when necessary, it promotes the adoption of healthy eating habits before pregnancy, and it recognizes and provides adequate intervention for micronutrient deficiencies [69].

Various studies performed with Spanish pregnant women have demonstrated that the diet followed by pregnant women is not totally adequate. Thus, Izquierdo-Guerrero [41], in his research with pregnant women from Madrid, finds a high protein and fat consumption, especially saturated fat, and a deficiency in micronutrients, which do not meet the recommended intakes (IR). On the other hand, Jardí et al. [32] determined that the consumption of red and processed meat and cakes and pastries exceeded recommendations whilst the consumption of healthy food decreased from the first trimester until after the postpartum period. 

Two studies performed by Ruiz [70] and Izquierdo-Guerrero [41] asserted that older women have a greater consumption of sugars, lipids, and MFA; similar data to that obtained in our study. Likewise, Izquierdo-Guerrero [41] did not find significant associations with income and CH consumption, unlike our study, which found that women with the lowest income consumed greater CH. On the other hand, according to Mohatar-Barba [71], Muslims usually eat less protein, which is similar to our results. The scarcity of studies that link sociocultural factors and diet quality in pregnant women is noteworthy. For this reason, we can consider that this is a novel study, as it is one of the first that links these variables. 

Among the study’s limitations we should highlight its cross-sectional design since it does not allow cause–effect relationships to be established among the variables. This study considers the eating habits of pregnant women, but it does not collect information about diet quality before conception and during the postpartum period, something that is considered important for correct monitoring. It should also be mentioned that the only factor not included in this study is tobacco consumption, which is usually considered when assessing lifestyles. As an incidental non-probability sampling is performed, it does not cover a significant representation of all the cultures found in Melilla. The sample quality can be improved by increasing the number of participants and choosing representatives from the different cities of Spain. On the other hand, the incorporation of nutrition education content into the educational system should be addressed, with the aim of fighting against inadequate eating habits that may cause long-term health problems. Finally, it should be mentioned that there was difficulty in finding studies on pregnant women that link diet quality with other sociodemographic factors.

With a view to the future, it is recommended that the first nutritional interventions are implemented in the preconception period, since this influences the state of the mother’s health, in addition to having an influence on the results of the pregnancy [30,72,73]. Performing an educational nutritional intervention as a pilot project should also be considered to be able to assess its subsequent implementation and improve the proposal’s design. Finally, another important aspect to consider is performing a stratified probability sampling. This would achieve a more representative sampling of Melilla’s population.

## 5. Conclusions

The results obtained in this study reveal that, in general, the caloric and lipid profiles of pregnant women in the city of Melilla do not meet the healthy recommendations established for the Spanish population. They consume too many proteins and fats, score high on SFA, and do not achieve CH recommendations as their diet contains twice the amount of sugar recommended. Likewise, they do not meet the recommendations for the intake of calcium, iron, magnesium, zinc, potassium, FA, and vitamins A, D, and E.

Furthermore, there are certain factors that may influence said intakes, such as religion, age, income, and marital status. Nevertheless, residency showed no association.

Regarding religion and marital status, Muslims and pregnant women with partners show a lower protein consumption. On the other hand, women with lower income consumed greater CH, and those with the lowest level of education consumed more simple sugars. Finally, older women consumed more lipids, specifically MFA.

## Figures and Tables

**Table 1:** Features of study sample (N = 306).

	Total (n = 306)
Residence	
Yes	255 (83.3)
No	51 (16.7)
Place of birth	
Melilla	196 (64.1)
Morocco	75 (24.5)
Other provinces	35 (11.4)
Number of children	
None	117 (38.2)
1–2	152 (49.7)
>2	37 (12.1)
Marital status	
Single	38 (12.4)
Couple/Married	265 (86.6)
Separated/Divorced	3 (1)
Religion	
Muslim	207 (67.6)
Christian	87 (28.4)
Other	12 (3.9)
Education	
No education	23 (7.5)
Primary and secondary	112 (36.6)
Baccalaureate/Vocational training (FP)	93 (30.4)
University/Postgraduate	78 (25.5)
Income	
EUR <500	12 (3.9)
EUR 501–1000	101 (33)
EUR 1001–2000	113 (36.9)
EUR 2001–5000	74 (24.2)
EUR >5001	6 (2)

**Table 2:** Energy, macronutrients, fiber, and cholesterol according to the gestational period (median and interquartile range).

Gestation Period		
Sample (N = 306)	1–20 Weeks (n = 145)	>20 Weeks (n = 161)
	Median (IQR)	Median (IQR)
Energy (Kcal)	1767.44 (632.01)	1898.49 (726.25)
Proteins (g)	72.41 (29.53)	74.54 (33.45)
Total CH (g)	196.95 (73.78)	216.14 (96.94)
Sugars (g)	76.56 (51.10)	85.96 (52.76)
Starch (g)	120.90 (59.02)	130.33 (61.19)
Total fat (g)	79.58 (38.03)	78.47 (38.63)
SFA (g)	22.79 (15.79)	23.85 (15.46)
MFA (g)	37.11 (19.70)	36.53 (17.05)
PFA (g)	9.39 (7.11)	8.76 (4.97)
Fibre (g)	12.86 (10.40)	15.62 (11.09)
Cholesterol (mg)	264.44 (273.45)	278.98 (240.48)

**Table 3:** Caloric and lipid profiles of the diet according to gestation period. Values in means (SD).

	Sample (N = 306)	Gestation Period		
		(1–20 Weeks) (n = 145)	(>20 Weeks) (n = 161)	*p* *
Caloric profile (Kcal supplied, %)				
Proteins	16.29 ± 4.25	16.13 ± 3.91	16.44 ± 4.54	0.517
Lipids	38.36 ± 7.17	39.54 ± 7.75	37.30 ± 6.45	0.006
Carbohydrates	45.46 ± 8.48	44.66 ± 9.37	46.18 ± 7.54	0.120
Simple sugars	18.60 ± 7.02	18.68 ± 7.92	18.54 ± 6.11	0.863
Caloric profile (Kcal supplied, %)				
MFA	18.26 ± 5.07	19.04 ± 5.64	17.55 ± 4.40	0.010
PFA	4.93 ± 2.01	5.21 ± 2.13	4.67 ± 1.86	0.018
SFA	11.53 ± 3.20	11.71 ± 3.21	11.37 ± 3.20	0.362

* Statistical test to determine significance: Mann–Whitney U test.

**Table 4:** Contribution of macronutrients and sugars to the TCV (caloric profile) (frequencies and percentages).

Gestation Period		
	1–20 Weeks (n = 145)	>20 Weeks (n = 161)
Proteins		
<10%	6 (4.1)	3 (1.9)
10–15%	60 (41.4)	65 (40.4)
>15%	79 (54.5)	93 (57.8)
Lipids		
<30%	10 (6.9)	19 (11.8)
30–35%	32 (22.1)	36 (22.4)
>35%	103 (71)	106 (65.8)
Carbohydrates		
<50%	106 (73.1)	114 (70.8)
50–55%	24 (16.6)	34 (21.1)
>55%	15 (10.3)	13 (8.1)
Simple Sugars		
<10%	18 (12.4)	13 (8.1)
>10%	127 (87.6)	148 (91.9)

**Table 5:** Contribution of the different fatty acids (MFA, PFA, and SFA) to the TCV (Lipid profile) (frequencies and percentages).

		Gestation Period	
		(1–20 Weeks) (n = 145)	(>20 Weeks) (n = 161)
SFA			
	Less than 10%	49 (33.8)	63 (39.1)
	More than 10%	96 (66.2)	98 (60.9)
MFA			
	Less than 20%	83 (57.2)	117 (72.7)
	20–25%	47 (32.4)	37 (23)
	More than 25%	15 (10.3)	7 (4.3)
PFA			
	Less than 5%	81 (55.9)	113 (70.2)
	5–10%	59 (40.7)	45 (28)
	More than 10%	5 (3.4)	3 (1.9)

**Table 6:** Mean intake of minerals and vitamins according to gestation period and recommendations (median and interquartile range).

Gestation Period				
Sample (N = 306)	1–20 Weeks (n = 145)		>20 Weeks (n = 161)	
	Median (IQR)	Reference *	Median (IQR)	Reference *
Minerals				
Ca (mg)	637.33 (481.71)	1000	815.11 (440.21)	1300
Fe (mg)	10.65 (4.54)	18	12.07 (5.48)	18
I (mg)	241.91 (273.91)	110	270.26 (238.97)	135
Mg (mg)	225.77 (90.81)	330	248.52 (112.48)	450
Zn (mg)	8.25 (4.47)	15	9.09 (4.13)	20
K (mg)	2499.71 (1100.24)	3500	2867.59 (1302.08)	3500
P (mg)	1109.40 (469.02)	700	1234.89 (560.23)	700
Vitamins				
B1 (mg)	0.89 (0.43)	0.9	0.96 (0.47)	1.0
B2 (mg)	1.38 (0.71)	1.4	1.58 (0.81)	1.6
Niacin eq. (mg)	27.36 (13.46)	15	27.07 (14.99)	17
B6 (mg)	1.55 (0.86)	1.6	1.87 (1.11)	1.9
FA (ug)	295.80 (198.86)	600	332.11 (197.78)	600
B12 (ug)	3.33 (2.96)	2	4.21 (2.92)	2.2
C (mg)	90.42 (107.54)	60	103.47 (89.20)	80
A: Retinol eq. (ug)	597.41 (626.15)	800	575.93 (577.48)	800
D (ug)	0.93 (1.94)	15	1.20 (2.91)	15
E (mg)	4.10 (3.43)	12	4.01 (3.73)	15

* Source: Moreiras et al. [40].

**Table 7:** Relationship between caloric profile and sociodemographic variables.

	Caloric Profile								
	CH		Proteins					Lipids	
	B	SE	β	B	SE	β	B	SE	β
Constant	49.43 ***	4.70	-	20.926 ***	2.34	-	30.88 ***	4.28	-
Resident	−0.690	1.369	−0.030	−0.086	0.680	−0.098	0.472	1.180	0.025
Age	−0.132	0.094	−0.086	−0.024	0.047	−0.032	0.142 *	0.080	0.109
Marital status	0.192	1.523	0.008	−1.416 *	0.753	−0.114	1.281	1.291	0.061
Religion	1.306	1.295	0.073	−1.209 *	0.638	−0.133	0.110	1.102	0.007
Education	1.109	1.066	0.065	0.761	0.529	0.042	−0.935	0.908	−0.065
Income	−2.778 *	1.266	−0.144	0.363	0.636	0.079	1.649	1.093	0.101

B: non-standardized coefficient; SE: standard error; β: Standardized coefficient. * *p* < 0.05; *** *p* < 0.001.

**Table 8:** Relationship between lipid profile and sociodemographic variables.

	Lipid Profile											
	MFA			PFA			SFA			Simple Sugars		
	B	SE	β	B	SE	β	B	SE	β	B	SE	β
Constant	13.59 ***	2.84	-	3.71 **	1.122	-	9.83 ***	1.78	-	13.06 ***	5.19	-
Resident	−0.378	0.823	−0.028	0.382	0.332	0.071	0.362	0.527	0.042	−0.607	1.146	−0.032
Age	0.148 **	0.056	0.161	0.022	0.022	0.061	−0.007	0.035	−0.013	−0.004	0.078	−0.003
Marital status	0.024	0.917	−0.002	0.019	0.341	0.003	0.748	0.542	0.081	0.619	1.269	0.030
Religion	0.234	0.774	0.021	0.082	0.242	0.021	−0.163	0.384	−0.026	0.931	1.076	0.062
Education	−0.465	0.642	−0.045	−0.172	0.148	−0.079	−0.378	0.235	−0.109	−2.820 *	1.582	0.106
Income	0.766	0.772	−0.067	−0.135	0.158	0.060	0.490	0.251	0.136	−1.395	1.060	−0.087

B: non-standardized coefficient; SE: standard error; β: Standardized coefficient. * *p* < 0.05; ** *p* < 0.01; *** *p* < 0.001.

## Data Availability

The data presented in this study are available on request from the corresponding author.
